# Use of population approach non-linear mixed effects models in the evaluation of biosimilarity of monoclonal antibodies

**DOI:** 10.1007/s00228-016-2101-6

**Published:** 2016-08-11

**Authors:** Joannes A. A. Reijers, T. van Donge, F. M. L. Schepers, J. Burggraaf, J. Stevens

**Affiliations:** Centre for Human Drug Research (CHDR), Zernikedreef 8, 2333 CL Leiden, The Netherlands

**Keywords:** Biosimilarity, Population pharmacokinetic modelling, Pharmacokinetics, Biological, Trastuzumab, Non-linear kinetics

## Abstract

**Purpose:**

Population pharmacokinetic analyses (PPK) have been used to establish bioequivalence for small molecules and some biologicals. We investigated whether PPK could also be useful in biosimilarity testing for monoclonal antibodies (MAbs).

**Methods:**

Data from a biosimilarity trial with two trastuzumab products were used to build population pharmacokinetic models. First, a combined model was developed and similarity between test and reference product was evaluated by performing a covariate analysis with trastuzumab drug product (test or reference) on all model parameters. Next, two separate models were developed, one for each drug product. The model structure and parameters were compared and evaluated for differences.

**Results:**

Drug product could not be identified as statistically significant covariate on any parameter in the combined model, and the addition of drug product as covariate did not improve the model fit. A similar structural model described both the test and reference data best. Only minor differences were found between the estimated parameters from these separate models.

**Conclusions:**

PPK can also be used to support a biosimilarity claim for a MAb. However, in contrast to the standard non-compartmental analysis, there is less experience with a PPK approach. Here, we describe two methods of how PPK can be incorporated in biosimilarity testing for complex therapeutics.

**Electronic supplementary material:**

The online version of this article (doi:10.1007/s00228-016-2101-6) contains supplementary material, which is available to authorized users.

## Introduction

During the past decades, many biotherapeutics have been marketed, mostly for use in the field of oncology and rheumatology. Although efficacious, high costs often limit the availability of these therapies or greatly burden the health care system. For example, treatment of a rheumatologic US patient with biologics costs on average $20,000 to $30,000 annually [1], and a single cycle of bevacizumab or other monoclonal antibody (MAb) can cost $2000 or more [2]. In 2014, the top 20 in global medicinal product sales contained 10 biotherapeutics, generating revenues between 4.4 and 11.8 billion dollar each [3]. Because of the growing number of patent expirations for the original biotherapeutics, it is expected that research of biosimilars will increase.

A first requirement for registration of the novel compound is to establish pharmacokinetic ‘biosimilarity’. Although the terminology slightly differs between the regulatory agencies, all agree on the basic concept of biosimilarity, which is that the novel (‘test’) compound should be highly similar to its originator (‘reference’) in terms of quality, efficacy and safety and that any remaining difference should be clinically insignificant [4–6].

Notwithstanding specific criteria for biotherapeutics, often, parts of guidelines for establishing bioequivalence—not biosimilarity—between chemically derived substances (‘small molecules’) are applied. These guidelines require that similarity should be demonstrated for key pharmacokinetic parameters, most commonly area under the concentration-time curve (AUC) and maximum concentration (*C*_max_), based on predefined acceptance limits at the highest dose level used. According to an evaluation by the World Health Organisation, studies proving biosimilarity generally use the 80–125 % equivalence range due to lack of specific acceptance criteria for biotherapeutics [4].

Although it is widely recognised that a non-compartmental analysis (NCA) is less appropriate when dealing with complex pharmacokinetics, it is still the most commonly used analytical method for demonstrating biosimilarity. Mentré’s group has extensively studied the use of population pharmacokinetic techniques in bioequivalence testing and found that it yielded similar results, with the modelling approach leading to a better understanding of the underlying biological system and the NCA being a relatively easy approach that does not require modelling and whose results can be used in a statistical analysis. The same was found for two biologicals, somatropin and epoetin-α [7–9].

We investigated whether a population pharmacokinetic analysis (PPK) could also be useful in bioequivalence testing for monoclonal antibodies (MAbs), which display complex elimination mechanisms, including non-linear routes, and have a plasma half-life of one to multiple weeks. Two approaches in modelling PK data were studied. First, we developed a combined model built on all available data for both the test and reference product and tested whether adding product (test/reference) as a covariate would improve the model, indicating non-similarity. Second, we developed separate models, one for test and one for reference product. This approach does not assume similarity as a starting point and allows comparison of the model structures and parameters. For this exercise, we chose the humanised MAb trastuzumab, which targets the HER2 receptor.

## Methods

### Study population and treatment

Data was gathered in a phase I randomised, single-dose, parallel group bioequivalence study, preceded by a placebo-controlled dose escalation part [10]. In this study, 110 male volunteers, aged 18–45 years, who were deemed healthy after a full medical screening, received trastuzumab in 250 mL 0.9 % NaCl as an intravenous infusion over 90 min. Two trastuzumab products were administered: the biosimilar product (test, T), codenamed FTMB (Synthon BV, Nijmegen, The Netherlands) and the EU-licenced product (reference, R), marketed as Herceptin®.

Studied dose levels of the test product in the dose escalation part were 0.5 mg/kg (*n* = 6), 1.5 mg/kg (*n* = 6) and 3 mg/kg (*n* = 6). The bioequivalence part consisted of 92 participants, who randomly received test (*n* = 46) or reference (*n* = 46) product at 6 mg/kg.

Based on the trastuzumab content of the used test and reference product vials, the actual dose levels were determined to be 0.49, 1.48, 2.96 and 5.96 mg/kg for T and 6.44 mg/kg for R.

### Bioanalyses

Trastuzumab was quantitated in serum samples collected pre-dose and at 45 min, 1.5 h, 2 h, 3 h, 4 h, 5 h, 6 h, 8 h and 24 h, and at 2, 4, 8, 14, 21, 28, 35, 42, 49 and 63 days after start of administration. A detailed description of the assay is given by Wisman [10]. The lower limit of quantification (LLOQ) was 0.060 μg/mL. All pre-dose trastuzumab concentrations <LLOQ were set to zero prior to analysis. Post-dose concentrations below LLOQ were not included. A serum sample for the quantification of serum HER2 extracellular domain (ECD) was collected prior to administration. This assay had a LLOQ of 0.50 ng/mL.

In the original clinical study protocol, the sample at day 63 was not collected for PK analysis and hence not included in the previously reported NCA results [10]. However, as this sample provided valuable insight in the non-linear clearance of trastuzumab, it was included in the analyses reported in this paper.

### PPK

#### General modelling approach

Population pharmacokinetic analysis (PPK) followed a step-wise approach. First, a general model for trastuzumab, hereafter referred to as ‘combined model’, was developed based on all available PK data for both test and reference product, including dose levels of the dose escalation part (0.49, 1.48 and 2.96 mg/kg). To investigate potential bias in the PK models due to analysing test and reference products simultaneously, PK models were also developed for the test (model T) and reference product (model R) separately and included only data from subjects who were exposed to 6 mg/kg test or reference product. These are hereafter referred to as ‘separate models’. The separate models were developed in parallel in order to maintain a structurally similar model for the test and reference product. Consequently, the model was only adopted if the corresponding model in the other treatment arm was preferred over its parent as well.

#### Model development

Model development was performed using non-linear mixed effects modelling (NONMEM 7.2.0, Icon Development Solutions, Ellicott City, MD, USA) and closely followed the FDA and EMA guidelines for PPK [11, 12]. Models were built under ADVAN 13 with tolerance (TOL) 9, and the first-order conditional method with interaction (FOCE-I) was used for parameter estimation. NONMEM reports an objective function value (OFV), which is the −2·log likelihood. Model hypothesis testing used the likelihood ratio test under the assumption that the difference in OFV is chi-square distributed with degrees of freedom being determined by the number of additional parameters in the more complex model. Hence, with a decrease in OFV of ≥7.88 points (*p* < 0.005), the model is preferred over its parent model. Also, model performance was evaluated by means of goodness-of-fit plots, using the software package R.

Several structural models with two or three compartments including combinations of linear and non-linear clearance were fitted to the data to determine the best structural model. Log-normal distribution of the between-subject variability (*η*) was assumed, and several residual error (*ε*) structures were tested (proportional, additive and combined).

Potential covariate correlations, defined as a significant Pearson’s product-moment correlation coefficient (*p* < 0.01 and *r* ^2^ > 0.5), were tested in the model development, in linear and exponential manners, and incorporated based on improvement in model performance. Explored covariates included lean body weight (LBW) [13], weight (WT), body surface area (BSA) [14], height (HT), BMI, age, HER2 ECD concentration, dose and product.

#### Model evaluation and predictive performance

To evaluate the robustness and predictive performance of the developed model, a visual predictive check (VPC) with 500 simulations was performed [15]. Prediction intervals of 95 % were obtained by simulating the model results from the original data. Model evaluation was performed by calculating the coefficient of variation to derive the uncertainty in the parameter estimates of the model which was considered acceptable when lower than 50 %. Also, shrinkage, as defined by Karlsson [16], was calculated to exclude model misspecification; shrinkage less than 30 % was considered acceptable.

#### Individual pharmacokinetic profiles

Individual pharmacokinetic profiles were simulated in R (version 3.2.2, R foundation for statistical computing, Vienna Austria) using the individual participant’s model parameter estimates. Integration was performed from the start of administration until the time point when the concentration in the central compartment dropped below 0.01 μg/mL. For the simulations, the following integration intervals were used: 1 s from administration until 24 h, 1 min until day 80 and 1 day thereafter. The concentrations were stored at original sampling times and at every 5 min. Trastuzumab concentration at the start of administration was assumed to be 0 μg/mL.

### Comparison to NCA

For comparison to a standard NCA, AUCs were derived using model *simulated* (predicted) individual concentrations at the original sampling times. AUC from administration (time 0) to the time of the last concentration > LLOQ (AUC_last_) was calculated using the linear trapezoidal method. AUC extrapolated to infinity (AUC_inf_) based on the apparent terminal elimination rate constant was calculated as well.

Biosimilarity statistics were performed on AUC_inf_ or AUC_last_ of all participants who were exposed to 6 mg/kg, comparing T to R in an unpaired *t* test, using the software package R. AUCs were natural log (ln)-transformed prior to statistical analysis. The estimated difference in means and the corresponding 90 % confidence interval (CI) were back-transformed to obtain the relative geometric mean ratio (GMR) of T over R (T/R). These results were then compared to those calculated in a standard NCA.

To correct for the difference between actual (5.96 and 6.44 mg/kg) and labelled dose (6 mg/kg), a linear normalisation to 6 mg/kg was applied to the individual AUCs in the NCA. In the PPK, individual profiles were simulated with the actual and labelled dose. Both corrected and uncorrected AUCs were calculated and statistically compared.

## Results

### Population

Pharmacokinetic data were gathered from 110 healthy male volunteers, whose demographics are presented in Table 1. In total, 1247 serum trastuzumab concentrations were available for the test product (T), of which 143 were <LLOQ (64 pre-dose). In the 6 mg/kg test group, 60/906 observations were <LLOQ (46 pre-dose) and for the reference product (Herceptin®), 51/912 observations (44 pre-dose).

**Table 1:** Demographics

	Test 0.5 mg/kg	Test 1.5 mg/kg	Test 3.0 mg/kg	Test 6.0 mg/kg	Reference 6.0 mg/kg
Parameter	(*n* = 6)	(*n* = 6)	(*n* = 6)	(*n* = 46)	(*n* = 46)
Age (year)	26.9 (8.9)	33.0 (9.1)	23.4 (2.3)	26.0 (7.3)	24.1 (5.8)
Height (cm)	183 (12.0)	176 (6.5)	184 (3.3)	184 (7.5)	182 (6.2)
Weight (kg)	73.1 (12.6)	73.0 (8.7)	72.0 (7.5)	79.5 (11.2)	77.1 (10.2)
BMI (kg/m^2^)	21.7 (3.3)	23.5 (2.6)	21.2 (2.1)	23.4 (2.5)	23.2 (2.7)
Lean body weight (kg)	59.4 (8.4)	57.5 (5.1)	59.1 (3.8)	62.6 (6.6)	61.0 (5.6)
Body surface area (m^2^)	1.93 (0.21)	1.89 (0.13)	1.92 (0.10)	2.01 (0.17)	1.97 (0.15)
ECD (μg/L)	12.7 (1.8)	11.8 (2.1)	11.4 (1.5)	11.3 (1.8)	11.8 (1.8)

Mean (SD) demographics per treatment arm

*LBM* lean body, *BSA* body surface area, *ECD* HER2 extracellular domain

### Model development

#### First step: combined model

Initial exploration of the data suggested that a two- or three-compartment model would describe the data best. Based on the observed non-linear kinetics, Michaelis-Menten kinetics was incorporated, described in terms of maximum rate of elimination (*V*_max_), and the concentration where ½·*V*_max_ is reached (*K*_*M*_). Addition of a linear elimination pathway, defined by elimination rate constant (*k*_*e*_), significantly improved the model fit for both the two- and three-compartment model.

**Fig. 1:**
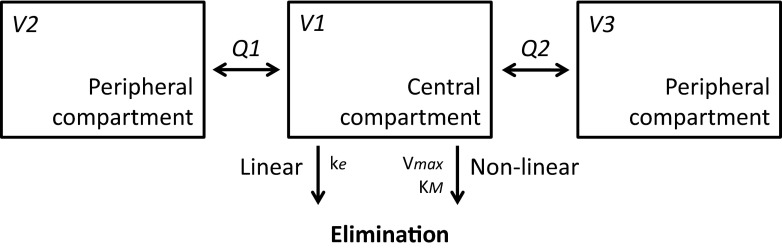
Schematic representation of the structural PK model with a parallel linear and non-linear elimination pathway. Linear elimination is described by an elimination rate constant (*k*_*e*_), and non-linear elimination is calculated as *V*_max_ *C* / (*K*_*M*_ + *C*) in which *V*_max_ is the maximum rate of elimination, *K*_*M*_ is the concentration which produces half of the *V*_max_ and *C* is the concentration. V1, V2 and V3 are the distribution volumes; Q1 and Q2 are the inter-compartmental clearances to the peripheral compartments

Adding the third compartment accounted for a delayed clearance effect. The three-compartment model, parameterised in terms of a central (V1) and peripheral volumes (V2, V3) of distribution and inter-compartmental clearances (Q1, Q2), resulted in a significant improvement compared to the two-compartment model. This was confirmed by an improved goodness-of-fit, especially for the lower doses of trastuzumab. Thus, the three-compartment model was considered superior over the two compartmental models (Fig. 1). A combined residual error structure (*ε*) proved best fit for purpose.

After identification of the structural model, individual estimates of random effects for between-subject variability were identified for the parameters V1, *K*_*M*_ and *k*_*e*_, with final coefficient of variation values of 14.8, 35.9 and 17.2 %, respectively. The residual coefficient of variation of the best model was 14.98 %. An omega block was required to correct for the parameter correlation between *K*_*M*_ and *k*_*e*_ in the model.

Significant correlations were found between lean body weight (LBW), body weight (WT), body surface area (BSA), height (HT) and body mass index (BMI) vs. V1, with correlation coefficients of 0.61, 0.55, 0.60, 0.54 and 0.28, respectively. Linear regression analysis of LBW vs. BSA resulted in a coefficient of 1 and for LBW vs. WT in 0.96. Furthermore, significant correlation coefficients were observed between BMI and *k*_*e*_ (0.60), between serum concentrations HER2 ECD and *k*_*e*_ (0.29), and between serum concentrations HER2 ECD and *K*_*M*_ (0.18).

Implementing LBW as a linear covariate on V1 (Online Resource Eq. 1) significantly improved the objection function value (OFV) and was added to the model. Incorporating other weight-related covariates (WT, HT and BMI) separately in the model did not result in a significant improvement compared to LBW; accordingly, they were not implemented in the model. Covariate analyses identified BMI as the one most significantly correlated to *k*_*e*_. Incorporating this covariate linearly on *k*_*e*_ (Online Resource Eq. 2) further improved the model, and BMI was thus added to the model. Incorporating HER2 ECD as a covariate did not improve the model fit. Interestingly, the model favours lean body weight as a size descriptor to scale trastuzumab dose compared to body weight, which is used clinically in dose calculation.

Adding trastuzumab drug product (test or reference) as a covariate to the model did not explain any relevant variability. A maximum decrease in OFV of only 5.80 points (*p* > 0.01) was observed when treatment was added as a covariate on *K*_*M*_. Thus, drug product as covariate did not significantly improve model fit. All PK parameter estimates obtained with the best fit of the models are listed in Table 2.

**Table 2:** Population PK parameters estimates from the full covariate model for trastuzumab

	Combined model	Separate model T	Separate model R
Parameter^a^	Estimate (CI)		
Fixed effects			
V1 (L)	3.28 (3.185–3.367)	3.59 (3.418–3.752)	3.13 (3.028–3.232)
V2 (L)	1.89 (1.325–2.457)	6.82 (−5.572–19.21)	44 (28.18–59.77)
V3 (L)	1.96 (1.736–2.179)	2.15 (1.858–2.443)	2.09 (1.929–2.244)
Q1 (L h^−1^)	2.91 (2.02–3.79) × 10^−3^	2.82 (1.081–4.566)	3.92 (3.58–4.25) × 10^−3^
Q2 (L h^−1^)	4.34 (3.66–5.01) × 10^−2^	3.75 (2.787–4.706)	4.67 (4.12–5.21) × 10^−2^
*V*_max_ (μg h^−1^)	178 (162.3–193.1)	172 (138.6–205.7)	127 (111–143.4)
*K*_*M*_ (μg L^−1^)	937 (759.6–1115)	995 (674.6–1316)	1440 (1189–1699)
*K*_*e*_ (h^−1^)	2.20 (2.02–2.38) × 10^−3^	1.95 (1.33–2.57) × 10^−3^	1.76 (1.62−1.9) × 10^−3^
Random effects	Estimate (CV%)		
Between-subject variability			
*ω* ^2^ V1	0.0217 (14.8)	0.0270 (16.5)	0.0122 (11.1)
*ω* ^2^ *V*_max_	–	0.0163 (12.8)	0.0347 (18.8)
*ω* ^2^ *K*_*M*_	0.121 (35.9)	–	–
*ω* ^2^ *k*_*e*_	0.0292 (17.2)	0.0355 (19.0)	0.0286 (17.0)
Residual error			
*σ* ^2^ proportional	0.0222	0.0207	0.0198
*σ* ^2^ additive	1520	3090	790

^a^Explanation of parameters is given in Fig. 1

*CI* confidence interval, *CV* coefficient of variation, *ω* ^*2*^ between-subject variance, *σ* ^*2*^ residual variance

Additionally, the rates of the linear and non-linear elimination pathway vs. serum concentration trastuzumab were calculated. At low serum concentrations of trastuzumab (<10 μg/mL), total elimination was almost independent of serum drug concentration, i.e. the non-linear elimination exceeded the linear elimination. At high concentrations, this pathway became saturated and the influence of non-linear elimination seemed negligible (Online Resource Fig. 3).

Also, a more complex mechanistic model approach was applied to characterise the distribution and clearance of trastuzumab: the target-mediated drug disposition (TMDD) model [17, 18]. Besides receptor and drug-receptor complex quantification, such models are able to provide information on binding affinity of the drug to the receptor. Fitting the TMDD model to our data proved difficult due to over-parameterization. A simplified approximation TMDD model approach with a dissociation constant *K*_*d*_ [19] still resulted in an incorrect fit and instability of the model, and the TMDD model approach was abandoned.

#### Second step: separate models

Model development of the separate models, including only data from participants who were exposed to 6 mg/kg, followed a similar approach as the combined model to ensure the structural similarity. For both trastuzumab products, a third compartment could be identified, as well as a linear and a non-linear route of elimination, described by Michaelis-Menten kinetics.

For the separate models, individual estimates of random effects for the between-subject variability were identified for the parameters V1, *V*_max_ and *k*_*e*_, with final coefficient of variation values in model T of 16.5, 12.8 and 19 %, respectively. The residual coefficient of variation of the best model was 14.5 %. In model R, the final coefficients of variation were 11.1, 18.8 and 17 %, with a residual coefficient of variation of 14.1 %.

Similarly to the combined model, the best model fit with the greatest reduction in OFV for both separate models was obtained by incorporating LBW as linear covariate on V1 and BMI on *k*_*e*_.

### Model evaluation and predictive performance

#### Combined model

**Fig. 2:**
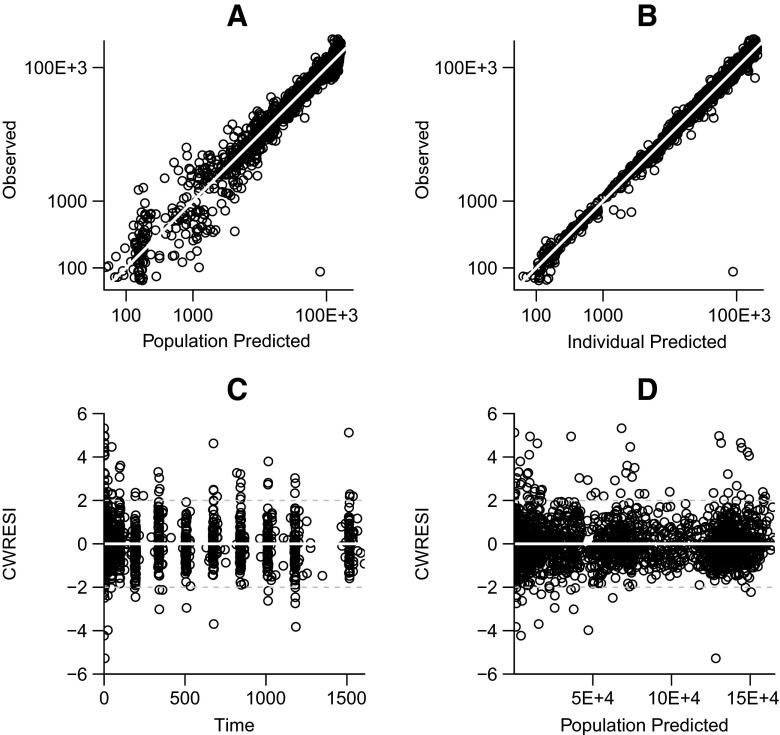
Goodness-of-fit plots combined model. Observed vs. population predicted concentration (**a**), observed vs. individual predicted concentration (**b**), conditional weighted residuals with interaction (CWRESI) vs. time (**c**), and conditional weighted residuals vs population predictions (**d**) of the combined model

Goodness-of-fit plots of the combined model (Fig. 2) show that all predictions lie around the line of unity. There was one outlier in the reference group, where one subject had a very low mid-infusion concentration of 0.088 μg/mL. Virtually all conditional weighted residuals with interaction (CWRESI) lie randomly scattered around zero without apparent bias.

The variability of the parameters V1, *K*_*M*_ and *k*_*e*_ on the Eta density histograms (Online Resource Fig. 4) seemed normally distributed around zero with acceptable coefficient of variation values, indicating correct description of the between-subject variability. Furthermore, no significant shrinkage was observed for parameters for which between-subject variability was identified (<8.04 %).

**Fig. 3:**
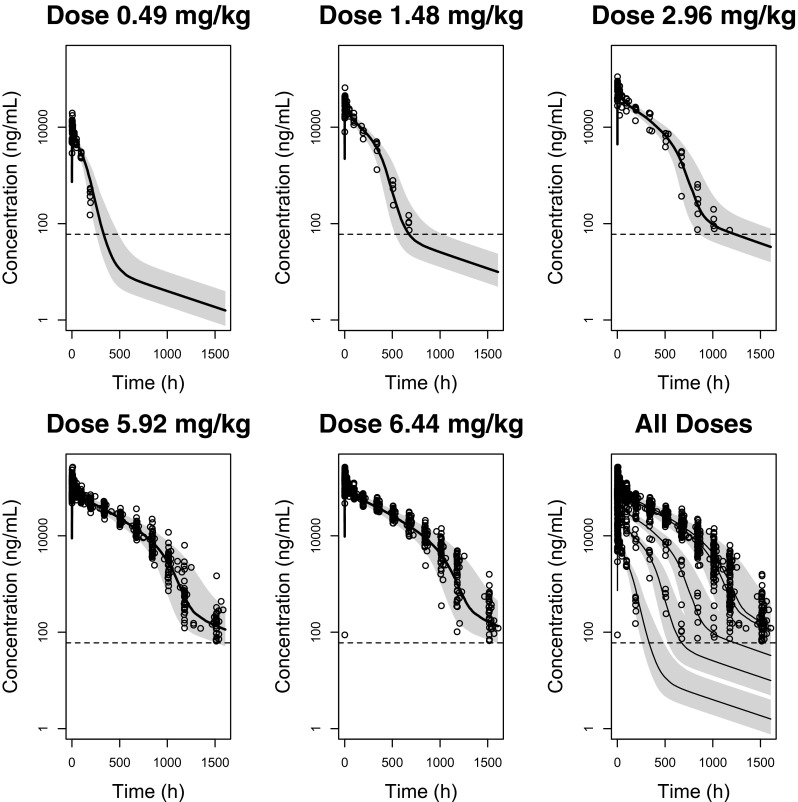
Visual predictive check (VPC). Visual predictive check (VPC) of the best combined model, conditioned per dose test product (0.49, 1.48, 2.96, 5.92 mg/kg) or reference product (6.44 mg/kg). The *dots* indicate the observations for the different trastuzumab doses administered, the *lines* are the typical predicted concentrations by the model for each dose and the *grey* area is the 95 % prediction interval (PI). The *dotted line* is the assay’s lower limit of quantification (LLOQ) for trastuzumab (0.060 μg/mL)

The visual predictive check (VPC) proofs good predictive performance (Fig. 3) of the combined model. For the doses >1.48 mg/kg, no signs of bias were apparent and most observations lay within the 95 % prediction interval (PI). Only for the lowest dose administered (0.49 mg/kg), a slightly higher prediction of the population mean was observed, especially in the lower concentration range. However, even for this dose group, most of the observations were within the 95 % prediction interval.

#### Separate models

The goodness-of-fit plots of the separate models (Online Resource Figs. 1–2) show that predictions lie around the line of unity and that the CWRESI are observed near the central line. No bias or trend in the model prediction could be determined. The shrinkage observed for the parameters for which between-subject variability was identified (V1, *V*_max_, *k*_*e*_) is not significant (<17.80 % for model T, <15.50 % for model R). Additionally, the variability on the eta density histograms (Online Resource Figs. 5–6) seemed normally distributed around zero.

The population PK parameter estimates from the full covariate model is presented in Table 2. When comparing parameter estimates, most parameter distributions overlap. The parameter estimates for V2 differ between model T and model R, but are in the same order of magnitude. However, Q1 and Q2 for model T were higher compared to model R. In contrast to the combined model, where between-subject variability was identified for V1, *K*_*M*_ and *k*_*e*_, in the separate models, these were found for V1, *V*_max_ and *k*_*e*_.

### Comparison to NCA

The geometric mean (GM) AUC_last_ obtained from the standard NCA was 1301 μg day^−1^ mL^−1^ for the test (T) and 1588 μg day^−1^ mL^−1^ for the reference (R) product. The AUC_last_ remained virtually unchanged when the same calculations were repeated with simulated concentrations, regardless of whether the combined model or the separate models were used (Table 3). Similar results were obtained with regard to AUC_inf_.

**Table 3:** AUC comparison actual dose

	GM		GM ratio (%)
AUC_last_	Test	Reference	T/R
NCA	1301	1588	81.91 (77.82–86.22)
Separate models	1300	1588	81.86 (78.08–85.82)
Combined model	1296	1588	81.59 (77.88–85.47)
AUC_inf_			
NCA	1311	1593	82.32 (78.17–86.69)
Separate models	1313	1591	82.54 (78.70–86.57)
Combined model	1300	1592	81.66 (77.93–85.56)

Geometric mean (GM) (μg day^−1^ mL^−1^) and GM ratio (%) with the 90 % confidence for the actual dose (test 5.92 mg/kg; reference 6.44 mg/kg) as derived by different methods per treatment arm

*T* test, *R* reference

The GM ratio (GMR) T/R with all AUC methods was 81.66–82.54 % with the lower limit (LL) of the associated 90 % confidence interval (CI) below the predefined equivalence boundary of 80 % (Table 3). Applying a linear correction to the NCA results caused the difference T–R in AUC_last_ and AUC_inf_ to decrease (GMR T/R 89.11–89.55 %, LL 90 % CI >84.66 %). Further reductions were achieved when an equal dosage of 6 mg/kg was *simulated* for both trastuzumab products, which affected the AUCs in the reference product arm more profoundly and increased the GMR with approximately 2 % point (Table 4).

**Table 4:** AUC comparison after dose correction

	GM		GM ratio (%)
AUC_last_	Test	Reference	T/R
NCA	1318	1479	89.11 (84.66–93.79)
Separate models	1323	1455	90.93 (86.72–95.35)
Combined model	1319	1443	91.41 (87.25–95.76)
AUC_inf_			
NCA	1329	1484	89.55 (85.03–94.30)
Separate models	1337	1457	91.74 (87.46–96.24)
Combined model	1324	1446	91.54 (87.37–95.92)

Geometric mean (GM) (μg day^−1^ mL^−1^) and GM ratio (%) with the 90 % confidence for the labelled dose (6 mg/kg) as derived by different methods per treatment arm. For the NCA results, a linear dose correction was applied; in the models, the labelled dose was used to simulate the individual profiles (see main body)

*T* test, *R* reference

Using the entire simulated profile, as opposed to only the simulated concentrations at the original sampling times, generally resulted in a small decrease of 1–2 % compared with the NCA for both AUC_last_ and AUC_inf_, with the exception of the AUC_inf_ calculated on profiles derived with model R, where an average increase of 1.7 % was observed (Online Resource Tables 1 and 2). Conversely, with the combined model, lower AUCs were obtained compared with the separate models for T only.

## Discussion

As long as generic products are being developed, controversy and scepticism regarding the claims of therapeutic equality have followed marketed bioequivalent products. Recently, Bate et al. [20] advocated that for the more complex pharmaceuticals, two allegedly bioequivalent drug products may not be interchangeable, which could have adverse consequences. MAbs are certainly among the most complex therapeutics, and establishing similarity to the reference product can thus be challenging.

For demonstrating pharmacokinetic biosimilarity in a human population, a NCA is virtually always performed and its results (AUC and *C*_max_) compared statistically, even though it is widely recognised that the NCA is less suitable for drugs with complex non-linear kinetics, as is the case for MAbs.

Population approach pharmacokinetic (PK) modelling and simulation techniques have been successfully applied to quantitatively describe the PK of MAbs in humans [21–26]. Such an approach has been applied in bioequivalence studies and also for biotherapeutics [9], where it was found to give indistinguishable results on the standard NCA parameters (AUC and C_max_), as was the case in our analysis. However, as was also argued by Dubois et al. [9], a PK model can provide valuable insight in the biological systems underlying the PK properties. Although the standard NCA-derived parameters, such as C_max_, AUC_inf_, terminal half-life, etc., may seem similar, the two drug products could behave quite differently in terms of PK, a feature that goes undetected in a NCA [27]. Furthermore, similar plasma concentrations do not invariably mean similar concentrations at the site of action.

Here, we describe two methods of incorporating PK modelling in biosimilarity research. The first approach is developing a model on all available data from both test and reference product(s) and carefully examining possible bias in one of the treatment groups. Testing for (statistically) significant differences between drug product can be done for all the model parameters via covariate analysis. Covariate testing follows a well-established statistical distribution that can be used for statistical inference [28, 29]. If no significant correlations can be identified between the drug products and if attempts to incorporate treatment as covariate in the model fail to improve it, the biosimilarity claim is supported.

The second method entails the development of different models, one for each test and reference product(s), which in contrast to a combined model does not assume similarity between test and reference product as a starting point. This method allows comparison of the model structure that should be identical for biosimilar products and of model parameters for both test and reference product.

Comparing different PK models inevitably reveals minor differences for which the clinical significance needs to be discussed. For example, in model T, the optimal inter-compartmental clearances (Q1 and Q2) were estimated to be a factor 10^2^–10^3^ higher than the corresponding parameters in the other models, while the striking dissimilarity did not seem to affect the descriptive properties of the overall profiles. However, as the (fictive) second and third compartments were not sampled, this finding merely reflects a mathematical solution to a rather complex problem and not necessarily a true (e.g. physiological or pharmacological) difference. Additionally, the higher dose administered for the reference product could have allowed a better characterisation of the terminal portion of the PK profile (elimination parameters), which also affects the estimation of remaining parameters such as Q1 and Q2.

This represents an important limitation of the second method, which may be of particular relevance when modelling PK data from two different populations separately. Unfortunately, pharmacokinetic biosimilarity of biotherapeutics is regularly investigated in trials of parallel design, because of the long half-life and the potential of anti-drug antibodies development, which could influence the pharmacokinetics [30]. Theoretically, all MAbs share common pharmacokinetic properties, e.g. small central volume of distribution, no renal excretion due to large molecular size, metabolism into amino acids and peptides, both specific (non-linear) and non-specific (linear) cellular uptake and degradation elimination mechanisms [31–35]. Thus, the remaining variability is probably determined by patient characteristics. When comparing the model parameters of the separate models, one of the most prominent differences is the population estimate for V1, which is unlikely caused by a difference between test and reference product.

The combined model equally well described the data, without bias in either the test or reference group. Adding trastuzumab drug product as covariate to the model could not explain any residual variability, which not only strongly supports the biosimilarity claim but also indicates that the difference in AUCs must be attributed to population characteristics.

From a regulatory perspective, another limitation of the second method is the lack of proper statistical inference testing on the model parameters. One might consider overlapping confidence intervals for parameter estimates indications for biosimilarity, but many parameters are related, so that for example a low inter-compartmental clearance may be ‘compensated’ in the model by a low volume of distribution. An extension of ‘bioequivalence statistics’ has been applied to model parameters by Wilkens et al. [36], although their method suffers from the aforementioned limitations as well.

Notwithstanding the limitations of PPK, it has several benefits over a NCA. Importantly, a PPK is not concerned with differences in administered doses. Although EMA allows a dose correction in the bioequivalence guideline (for chemically-derived products) if the difference exceeds 5 %, the NCA assumes linearity in its correction, which is not appropriate for MAbs, that display non-linear pharmacokinetics. Other benefits of PPK include the possibility to identify and thus correct for certain covariates and the relative robustness of a PPK against protocol deviations, with regard to timing of sample collection, missing samples, duration of intravenous administration and incomplete administration [8, 37].

Simulations with model R revealed that the two allowed extremes for protein content per batch (effective dose 5.28 and 7.2 mg/kg) would result in a 90 % CI for the GMR for AUC_last_ of 146.39–147.22 % in a cross-over design (*n* = 46). If such batch-to-batch variations are not considered relevant, then the consequences on the standard biosimilarity parameters may also be argued to be irrelevant.

With a PK model, multiple scenarios can be simulated within these extremes, which can be used to build the case that the test product achieves therapeutic drug concentrations, similar to the reference product, when administered according to a certain dosing regimen. This approach also circumvents some of the aforementioned limitations of direct comparison of two or more models. If a biomarker or pharmacological effect can be measured in the biosimilarity trial and incorporated in a pharmacokinetic-pharmacodynamic model (pharmacodynamic model), a relevant clinical target may be simulated and lend further support to a biosimilarity claim.

The NCA will most likely remain a gold standard in biosimilarity research, even for the complex MAbs. Nonetheless, the model approach can serve as an elegant add-on. Questions that need to be addressed before a PPK can fully substitute the NCA in demonstrating biosimilarity relate to selection of the most meaningful PK or pharmacodynamic parameter from the model, and the minimal population size to detect with sufficient statistical power relevant (model) differences.

Previously, the benefits of modelling and simulation have been proposed for proof of biosimilarity, to which this paper adds similar benefits for MAbs.

## Electronic supplementary material

ESM 1 (PDF 1081 kb)
