# Supplementary material for: Use of population approach non-linear mixed effects models in the evaluation of biosimilarity of monoclonal antibodies
Source: Eur J Clin Pharmacol. 2016 Aug 11;72(11):1343–52. doi: 10.1007/s00228-016-2101-6 (PMC5055907; doi:10.1007/s00228-016-2101-6)

Use of population approach nonlinear mixed effects models in the evaluation of biosimilarity of monoclonal antibodies

J.A.A. Reijers, T. van Donge, F.M.L. Schepers, J. Burggraaf, J. Stevens

Centre for Human Drug Research (CHDR), Leiden, The Netherlands

Correspondence:
Centre for Human Drug Research (CHDR)
Zernikedreef 8
2333 CL Leiden
The Netherlands
+31715246400 (phone)
+31715246499 (fax)
info@chdr.nl

Supplement

Equations

$$V1_i = \theta_{\text{pop}} \times \frac{LBW_i}{LBW_{median}} \times \exp(\eta_i) \quad (1)$$

$$k_{e,i} = \theta_{\text{pop}} \times \frac{BMI_i}{BMI_{median}} \times \exp(\eta_i) \quad (2)$$

where i, i^{th} individual; *V1*, volume of distribution, $k_{e,}$ elimination rate constant; $\theta_{population}$, population parameter estimate; η, inter-individual variability; *LBW*, lean body weight; *BMI*, body mass index.

Supplementary table 1
AUC comparison actual dose

	Test			Reference		
**AUC_{last}**	**AUC**	**%-change[a]**	**%-change[b]**	**AUC**	**%-change[a]**	**%-change[b]**
NCA	1318 (220)			1602 (220)		
Separate models						
Actual time	1315 (211)	100.0 (5.0)		1599 (190)	100.1 (4.5)	
Continuous time	1292 (206)	98.3 (4.9)		1571 (188)	98.4 (4.5)	
Combined model						
Actual time	1310 (204)	99.8 (5.4)	99.7 (1.1)	1599 (193)	100.1 (4.9)	100.0 (1.5)
Continuous time	1287 (199)	98.0 (5.2)	99.7 (1.1)	1571 (191)	98.4 (5.0)	100.0 (1.5)
**AUC_{inf}**						
NCA	1328 (225)			1607 (224)		
Separate models						
Actual time	1329 (216)	100.3 (5.2)		1602 (192)	100.0 (4.5)	
Continuous time	1312 (208)	99.0 (5.0)		1630 (201)	101.7 (4.7)	
Combined model						
Actual time	1315 (206)	99.3 (5.7)	99.1 (3.3)	1604 (195)	100.1 (4.9)	100.1 (1.5)
Continuous time	1298 (200)	98.1 (5.3)	99.1 (1.1)	1577 (193)	98.4 (5.0)	96.8 (1.2)

Mean (SD) AUCs (µg·day·mL^{-1}) and mean (SD) percentage change for the actual dose (test: 5.92 mg/kg; reference 6.44 mg/kg) as derived by different methods per treatment arm. AUCs of combined and separate models are compared to the NCA result (a); AUCs of combined model are compared to the AUCs of the separate model (b, actual time compared to actual time, continuous time to continuous time). For comparison to a standard NCA, AUC_{last} was calculated using model simulated (predicted) individual concentrations at the original sampling times (‘actual time’). Extrapolation to infinity (AUC_{inf}) was based on the apparent terminal elimination rate constant. Additionally, the AUCs were derived by integration (1 s intervals) of the simulated concentration-time profiles (‘continuous time’); AUC_{last} from the administration time to the last concentration used in the NCA, AUC_{inf} until infinity.

Supplementary table 2
AUC comparison after dose correction

	Test			Reference		
**AUC_{last}**	**AUC**	**%-change[a]**	**%-change[b]**	**AUC**	**%-change[a]**	**%-change[b]**
NCA	1335 (223)			1493 (205)		
Separate models						
Actual time	1338 (215)	100.5 (5.1)		1465 (176)	98.4 (4.5)	
Continuous time	1315 (210)	98.7 (5.0)		1439 (174)	96.7 (4.4)	
Combined model						
Actual time	1334 (208)	100.2 (5.4)	99.7 (1.1)	1453 (176)	97.7 (4.8)	99.2 (1.2)
Continuous time	1310 (202)	98.5 (5.3)	99.7 (1.2)	1427 (174)	95.9 (4.8)	99.2 (1.3)
**AUC_{inf}**						
NCA	1346 (228)			1497 (209)		
Separate models						
Actual time	1353 (220)	100.7 (5.2)		1467 (177)	98.3 (4.5)	
Continuous time	1335 (212)	99.5 (5.0)		1493 (185)	100.0 (4.7)	
Combined model						
Actual time	1339 (210)	99.8 (5.8)	99.1 (3.4)	1456 (177)	97.6 (4.8)	99.2 (1.2)
Continuous time	1322 (204)	98.6 (5.3)	99.1 (1.2)	1432 (175)	96.0 (4.8)	96.0 (1.0)

Mean (SD) AUCs ($\mu g \cdot day \cdot mL^{-1}$) and mean (SD) percentage change for the labelled dose (6 mg/kg) as derived by different methods per treatment arm. For the NCA-results a linear dose correction was applied; in the models the labelled dose was used to simulate the individual profiles (see main body). AUCs of combined and separate models are compared to the NCA result (a); AUCs of combined model are compared to the AUCs of the separate model (b, actual time compared to actual time, continuous time to continuous time). For comparison to a standard NCA, AUC_{last} was calculated using model simulated (predicted) individual concentrations at the original sampling times ('actual time'). Extrapolation to infinity (AUC_{inf}) was based on the apparent terminal elimination rate constant. Additionally, the AUCs were derived by integration (1 s intervals) of the simulated concentration-time profiles ('continuous time'); AUC_{last} from the administration time to the last concentration used in the NCA, AUC_{inf} until infinity.

Supplementary figure 1
Goodness of fit plot model T

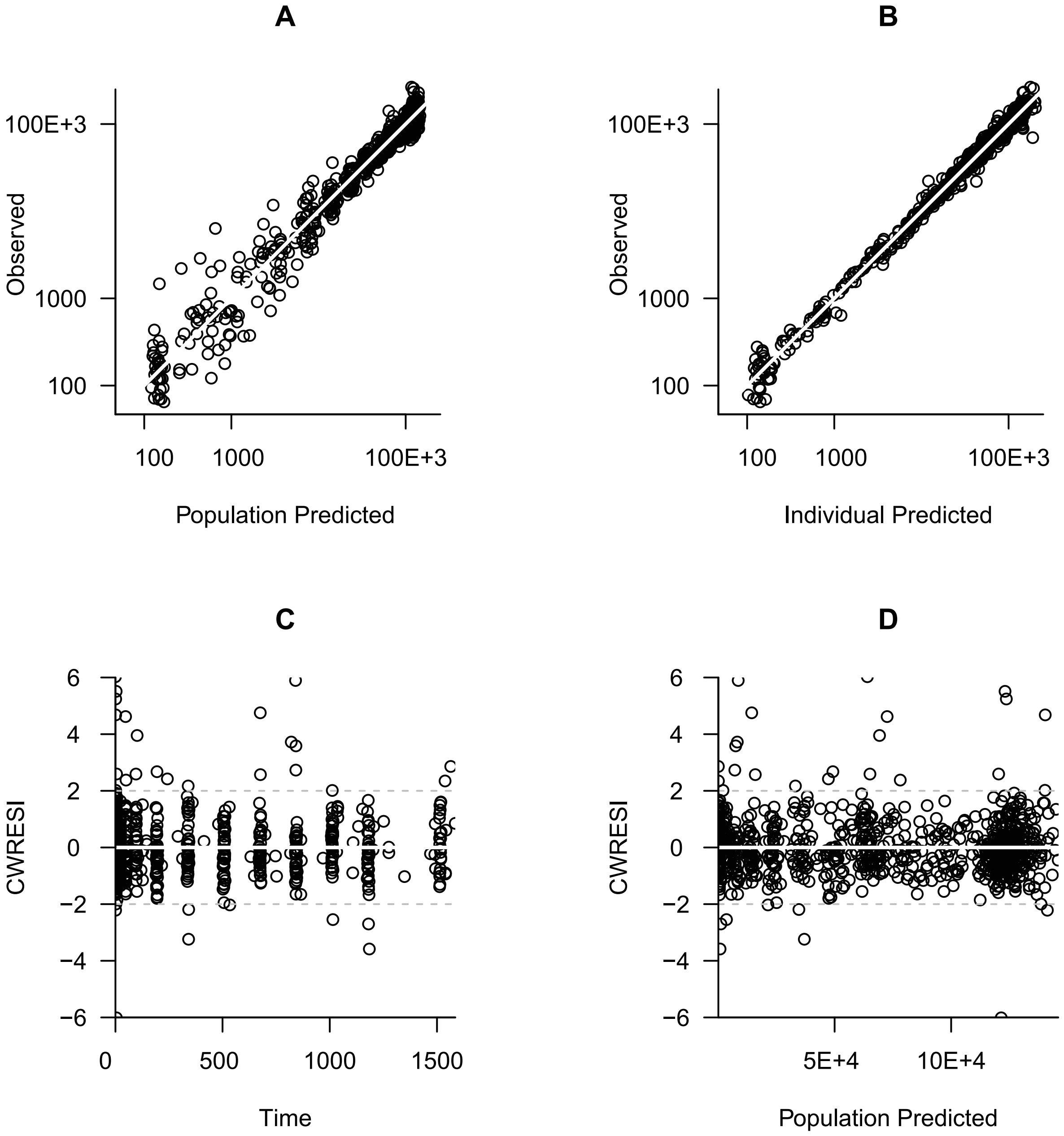

Observed *vs.* population predicted concentration (A), observed *vs.* individual predicted concentration (B), conditional weighted residuals with interaction (CWRESI) *vs*. time (C), and conditional weighted residuals *vs*. population predictions (D) of the separate model for the test product.

Supplementary figure 2
Goodness of fit plot model R

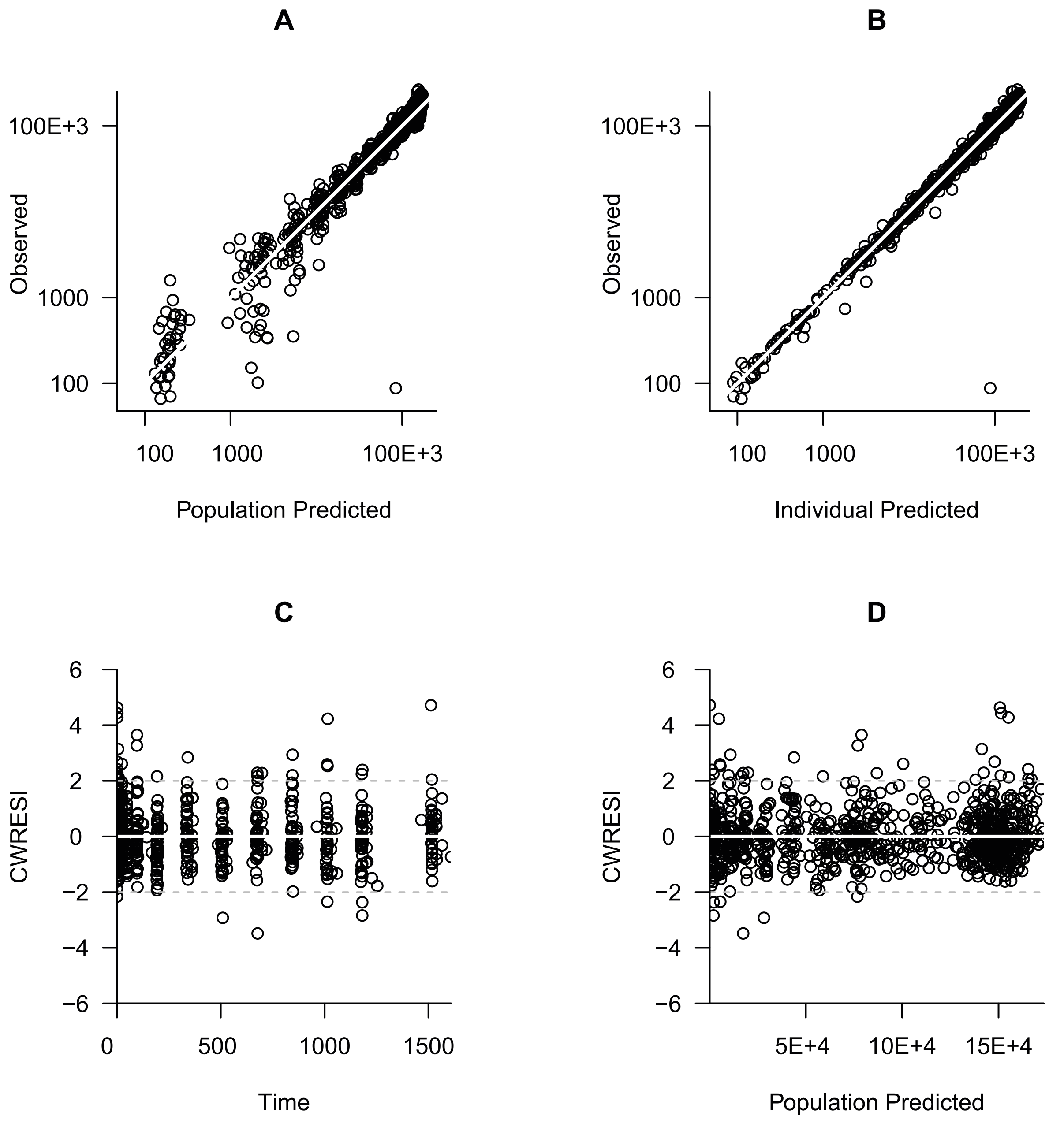

Observed *vs.* population predicted concentration (A), observed *vs.* individual predicted concentration (B), conditional weighted residuals with interaction (CWRESI) *vs*. time (C), and conditional weighted residuals *vs*. population predictions (D) of the separate model for the reference product.

Supplementary figure 3
Linear and non-linear clearance combined model

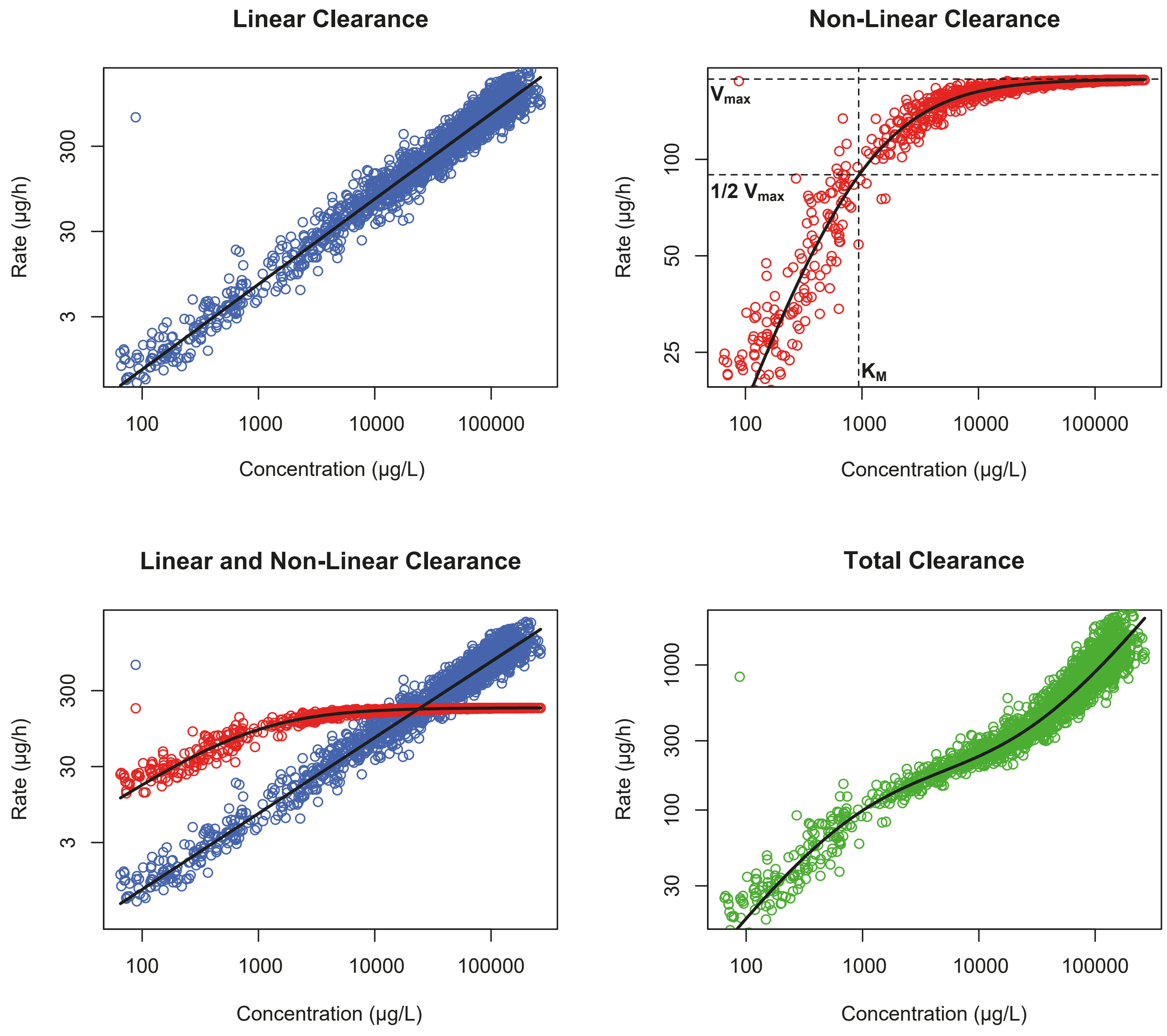

Supplementary figure 4
V1, K_M and k_e η density histograms for combined model

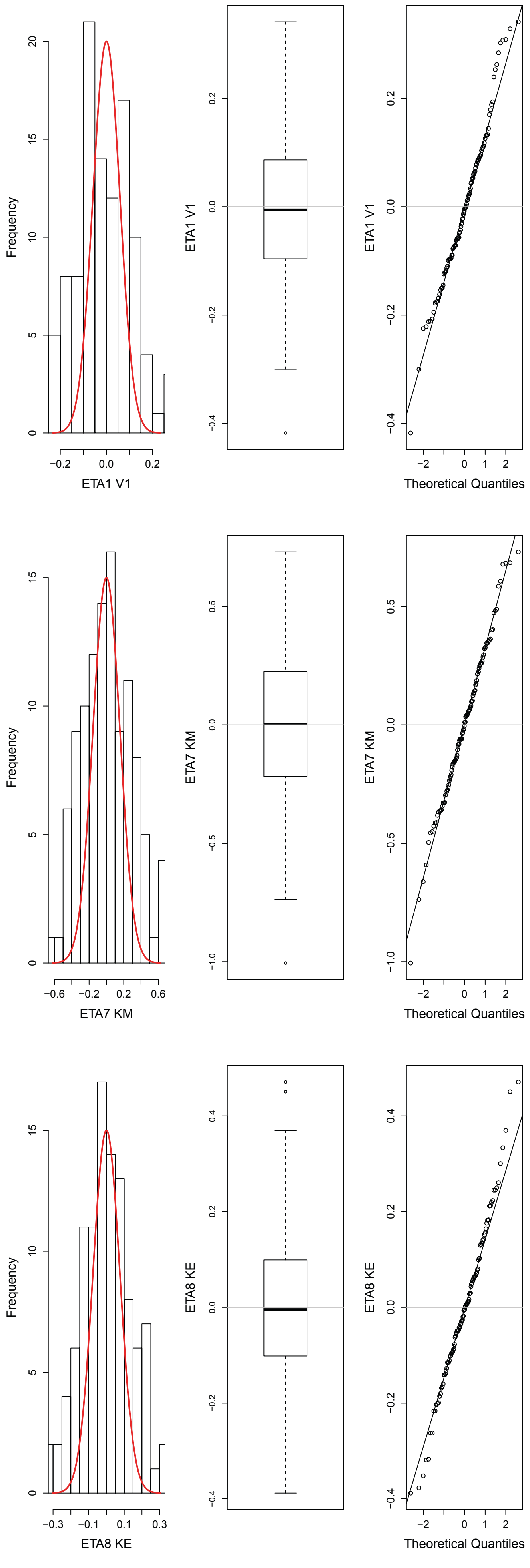

Supplementary figure 5
V1, V_{max} and k_e η density histograms for model T

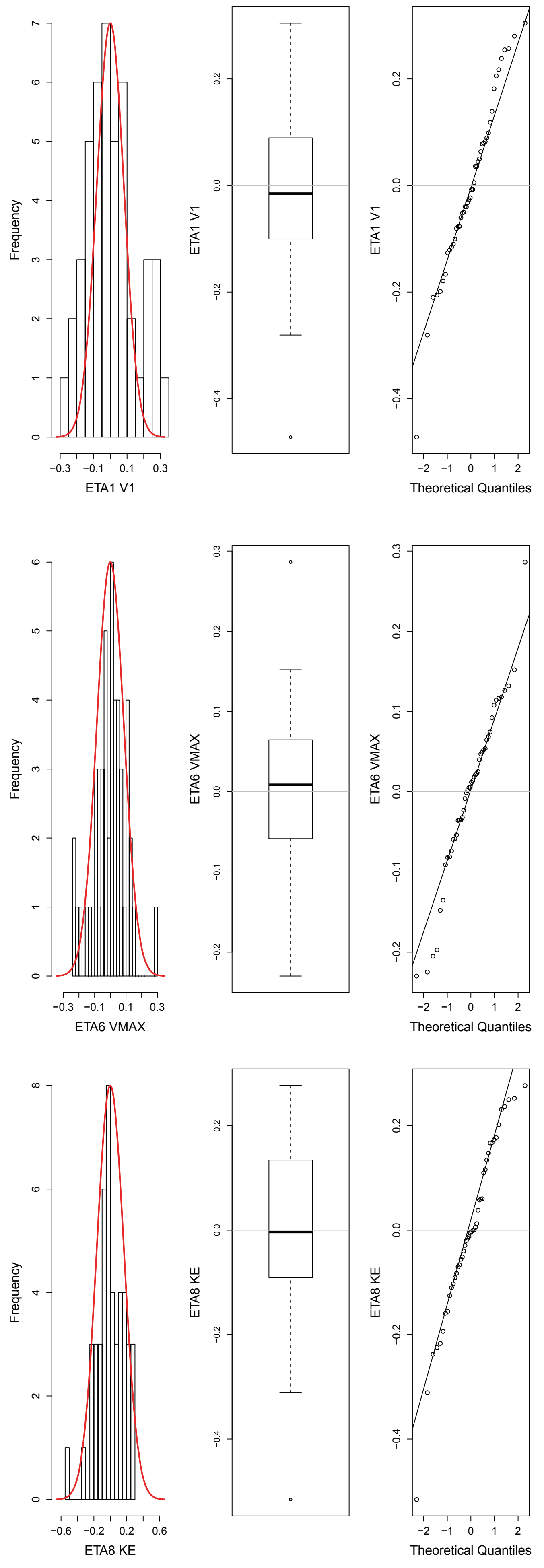

Supplementary figure 6
V1, V_{max} and k_e η density histograms for model R

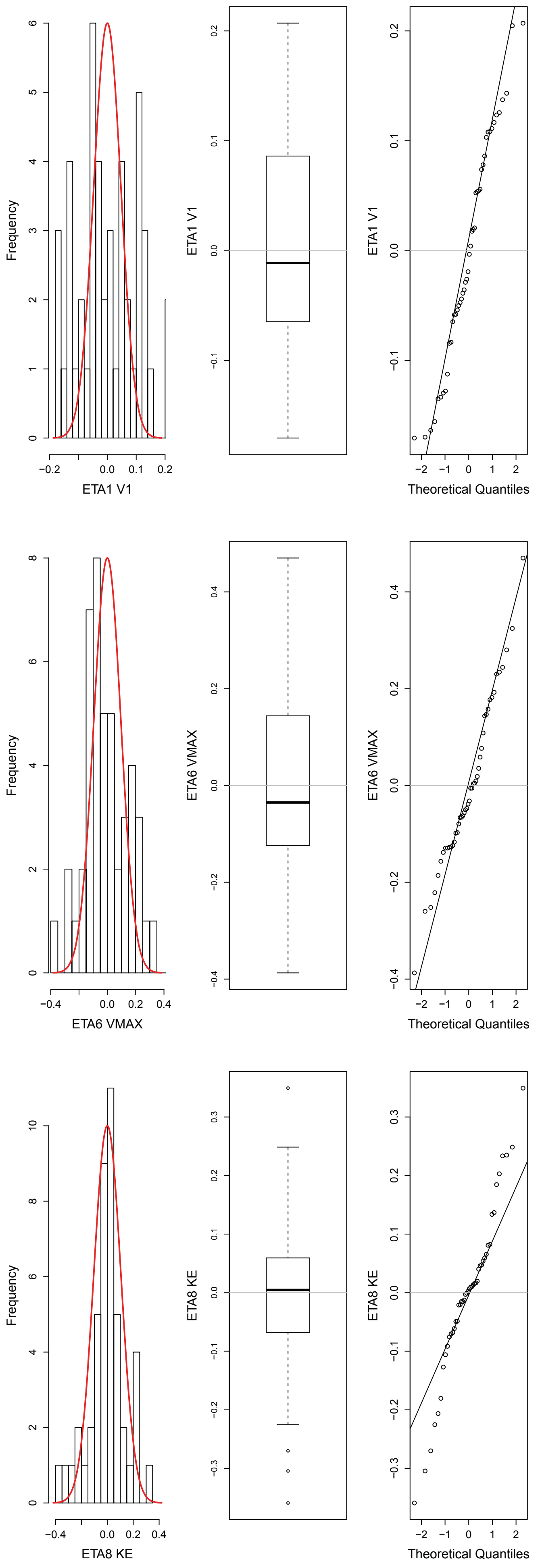

Supplement: Supplementary file 1 — (PDF 1081 kb) [file 228_2016_2101_MOESM1_ESM.pdf]
